# Dementia, a special supplement

**Published:** 2009-01

**Authors:** T. S. Sathyanarayana Rao, K. S. Shaji

**Affiliations:** Department of Psychiatry, JSS University, JSS Medical College, Mysore - 570004, India; 1 Department of Psychiatry, Medical College, Thrissur - 680596, Kerala, India

Dementia is fast emerging as a huge public health challenge in the developing world. We need to prepare ourselves to face this crisis. We must know more about various aspects of dementia and its management. Better understanding of dementia in developing countries is also likely to provide valuable new information on risk factors, genetics, and potential new treatments. That is why we chose “Dementia” as the theme.

We are pleased to present a supplement of Indian Journal of Psychiatry (IJP) on Dementia. Dementia being a neglected area in our country - both concerning the research and service, there are many invited articles from abroad. We hope this will act as an impetus to further the cause of dementia. A special issue on a specific topic and on this scale is first in the history of IJP. We have tried to get articles from many people who have contributed to the knowledge base. Most of them responded wholeheartedly and well in time. We are delighted to acknowledge their help and support. We remain deeply indebted to them.

We look forward to your feed back. Please send in your comments, criticisms and suggestions.

Yours sincerely

**T. S. S. Rao,**
Editor, Indian Journal of Psychiatry **K. S. Shaji,**
Guest Editor, Indian Journal of Psychiatry supplement on Dementia


